# Randomized Clinical Trial Comparing Insertion Torque and Implant Stability of Two Different Implant Macrogeometries in the Initial Periods of Osseointegration

**DOI:** 10.3390/medicina59010168

**Published:** 2023-01-14

**Authors:** Sergio Alexandre Gehrke, Guillermo Castro Cortellari, Gustavo Vicentis de Oliveira Fernandes, Antonio Scarano, Rafael Garcia Martins, Renata Moreira Cançado, Alfredo Mikail Melo Mesquita

**Affiliations:** 1 Department of Research, Bioface/PgO/UCAM, Calle Cuareim 1483, Montevideo 11100, Uruguay; 2 Instituto de Bioingenieria, Universidad Miguel Hernández, Avda. Ferrocarril s/n., 03202 Elche, Spain; 3 Department of Biotechnology, Universidad Católica de Murcia (UCAM), 30107 Murcia, Spain; 4 Department of Materials Engineering, Pontificia Universidade Católica do Rio Grande do Sul, Porto Alegre 90619-900, Brazil; 5 Periodontics and Oral Medicine Department, University of Michigan School of Dentistry, Ann Arbor, MI 48109, USA; 6 Department of Innovative Technologies in Medicine & Dentistry, University of Chieti-Pescara, 66100 Chieti, Italy; 7 Department of Implantology, Paulista University (UNIP), São Paulo 04026-002, Brazil

**Keywords:** clinical study, implant macrogeometry, implant stability, insertion torque, resonance frequency analysis

## Abstract

*Objectives*: The present study compared two implants with different macrogeometries placed in healed alveolar sites, evaluating the insertion torque (ITV) and implant stability quotient (ISQ) values at three different periods. *Methods*: Seventy patients with a total of 100 dental implants were allocated into two groups (*n* = 50 per group): DuoCone implants (DC group) that included 28 implants in the maxilla and 22 in the mandible, and Maestro implants (MAE group) that included 26 in the maxilla and 24 in the mandible. The ITV was measured during the implant placement, and the ISQ values were measured immediately at implant placement (baseline) and after 30 and 45 days. *Results*: The mean and standard deviations of the ITV were statistically significant (*p* < 0.0001), 56.4 ± 6.41 Ncm for the DC group and 29.3 ± 9.65 Ncm for the MAE group. In the DC group, the ISQs ranged between 61.1 ± 3.78 and 69.8 ± 3.86, while the MAE group presented similar values compared with the other group, ranging between 61.9 ± 3.92 and 72.1 ± 2.37. *Conclusions*: The value of implant insertion torque did not influence the ISQ values measured immediately after implant placement. However, the ITV influenced the ISQ values measured in the two initial periods of osseointegration, with implants installed with lower torques presenting higher ISQ values.

## 1. Introduction

Even though dental implants have high predictability, new micro and macrogeometries have been researched, developed, and commercialized. Recently, new implant designs aimed to accelerate the osseointegration process with macrogeometry modifications have been proposed [1,2,3]. These modifications were mainly based on the reduction in compression caused by the insertion of the implant in the bone bed with less pressure (lower insertion torque value).

Thereby, implant geometries and bone density have been considered the main factors involved in the degree of primary implant stability [4]. However, for many decades, high values obtained by the insertion torque were considered a success factor for the osseointegration of implants since the torque was directly related to the primary implant stability. However, recent studies have shown that lower torque can reduce trauma to bone tissue and benefit or facilitate the bone healing process without affecting the primary stability [1,5,6]. To reduce the insertion torque without affecting their stability, spaces for bone decompression must be created through healing chambers in the implant body or implants with large threads requesting the use of a drill with a larger diameter than the implant body to promote adequate stability [2,6]. For cases with poor bone quality, large-thread implant designs are highly desirable [4].

In the conditions described, the osteogenesis at the implant/bone interface can occur in two directions, with bone formation from the receptor site towards the implant (distance osteogenesis) and from the implant towards the adjacent bone, which is called contact osteogenesis [7,8,9]. There is evidence that contact osteogenesis has a 30% higher rate of bone formation than distant osteogenesis [10]. Changing the shape of the implants or the osteotomy used makes it possible to direct between these two types of bone formation during the initial stages of osseointegration [11,12]. In these spaces created between the bone tissue and the implant (healing chambers) [2,3,6], there is a predominance of contact osteogenesis. Implants combined with osteotomy are an alternative for creating healing chambers [6], in which both types of osteogenesis (contact and distance) may predominate.

On the other hand, the importance of the initial implant stability as a pre-requisite for the success of osseointegration and the long-term success of implant-supported restorations is a consolidated condition in implantology [13,14]. However, the level of stability depends on some factors, such as the quantity and quality of the local bone, implant design, and surgical technique applied [15,16,17,18,19], while the degree of secondary stability is dependent on changes that occur during the healing phase of the bone tissue, that is, on the cellular activity of resorption, neoformation, and mineralization at the bone-implant interface [20,21].

Otherwise, the possibility of measuring stability with the emergence of new specific devices for this purpose meant that the determination of the proper fixation of the implant no longer needed to be mechanical, that is, by the torque value [22,23]. Thus, through resonance frequency analysis (RFA), a noninvasive method, it is possible to measure the stability and presumed osseointegration of implants [22,23,24], and it is a valuable tool for establishing timing for implant loading. Clinically, RFA values have been correlated with changes in implant stability during osseous healing, the failure of implants to integrate, and the supracrestal dimensions of the implant [25].

Therefore, the objective of the present study was to measure the maximum insertion torque values (ITV) and the implant stability quotient (ISQ) values at three different time points after surgical placement (immediately, 30, and 45 days later) to evaluate the influence of dental implant macrogeometry on the early stability.

## 2. Materials and Methods

### 2.1. Patient Population and Distribution

A total of 70 patients were selected and included in this study, 38 women and 32 men, whose ages ranged from 23 to 68 years old. The present clinical study was submitted and approved by the Ethics Committee of the Paulista University (UNIP, São Paulo, Brazil) under the number 5,728,743. All procedures were performed by the same professional (SAG), who has extensive experience in implantology. Previously, all selected participants were informed about the conditions and the type of study that would be carried out. They completed and signed the informed consent according to the agreement of the Declaration of Helsinki of 1994. The Consolidated Standards of Reporting Trials (CONSORT) guidelines were followed during the manuscript preparation.

For the inclusion of participants, the current medical conditions of each patient, their ability to withstand the stress of the surgical procedure for implant placement, and their need to replace lost teeth in the maxilla or the mandible in healed alveolar sites were considered. All selected patients agreed to participate in the non-invasive study to measure implant stability based on resonance frequency analysis for 45 days. Cases in which immediate loading was indicated were not included in this study. Other patients excluded from this study were those with systemic alterations, such as diabetes, hypertension, or osteoporosis, oral pathologies of soft or hard tissues, and harmful oral habits, such as bruxism and smoking (>10 cigarettes per day). Local exclusion factors (intraoral) were considered for patients with uncontrolled or untreated periodontal disease, areas with little bone volume for implant insertion, and active infection in sites close to the implant installation area.

### 2.2. Implant Macrogeometries and Surface Treatment

**Figure 1:**
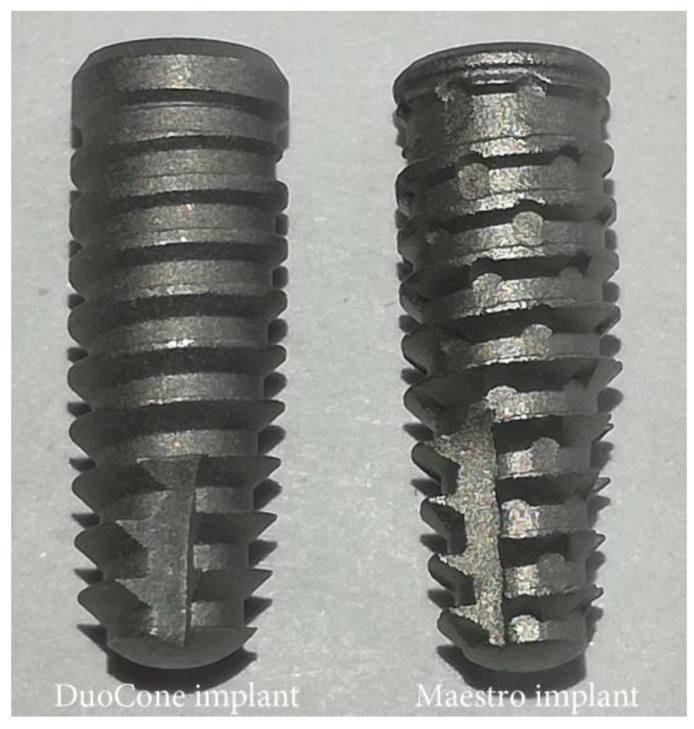
Representative image of both tapered implants with different macrogeometries used in the present study.

In the present study, two implant macrogeometries of Morse taper connection manufactured in Grade-4 titanium (Implacil De Bortoli, São Paulo, Brazil) were used, as described in the following: a DuoCone implant, which presents a regular conical macrogeometry with progressive trapezoidal threads and a cervical plane area of 1 mm; whereas, the Maestro implant shows a conical macrogeometry with progressive trapezoidal threads, a cervical plane area of 0.5 mm, and healing chambers between the threads. Figure 1 presents an image of the two implant macrogeometries.

All implants received an equal surface treatment by blasting with 50–100 µm TiO_2_ particles and were ultrasonically cleaned with an alkaline solution, washed in distilled water, and pickled with maleic acid (HO_2_CCH_2_CHOHCO_2_H). These treatments promote an absolute value of all profile points (Ra) of 0.87 ± 0.14 μm, root-mean-square of the values of all points (Rq) of 1.12 ± 0.18 μm, and the average value of the absolute heights of the five highest peaks and the depths of the five deepest valleys (Rz) of 5.14 ± 0.69 μm, as previously published by our research group [25].

### 2.3. Experimental Design

The patients were divided into two groups (*n* = 50 implants per group): 31 subjects for the DC group that received 28 implants in the maxilla and 22 implants in the mandible, and 39 subjects for the MAE group that received 26 implants in the maxilla and 24 implants in the mandible. All patients who needed more than one implant in different areas were included in the same group, and initially, they were all randomized (www.randomizer.org, accessed on 24 April 2022) and assigned to determine their group. Moreover, the following clinical information was collected: patient age, gender, implant location, implant macrogeometry, implant length, and implant diameter.

### 2.4. Surgical Procedure

Standard routine surgical procedures were applied. As a preoperative medication, all patients received Amoxicillin (875 mg orally twice daily or Clindamycin 600 mg if allergic to penicillin) for 5 days, with an initial dose (2 tablets) administered 2 h before surgery. The same surgeon, a specialist in implantology with many years of experience in this field, installed all implants. Firstly, patients were anesthetized with local infiltrative anesthesia using 2% Articaine (DFL Ltd., Rio de Janeiro, Brazil). A mucosal incision was then made up to the periosteal level, the full-thickness mucoperiosteal tissue was elevated, and the osteotomies were performed using the sequence of drills indicated by the manufacturer (in this case, the same for both implant models) for each implant diameter used. A total of 100 implants were installed, with diameters of 3.5 mm (*n* = 42) or 4 mm (*n* = 58) and lengths that ranged from 9 to 13 mm. The implant dimensions were selected based on the prior evaluation of each case.

For drilling, an implant motor and a 20:1 reduction contra-angle (Bien-Air Surgery SA, Le Noirmont, Switzerland) were used under external irrigation with a 0.9% saline solution. All implants were installed using surgical guides, and the wounds were sutured. All implants were installed using the motor at a rotation of 30 RPM; a torque value was obtained after implant placement. Ketoprofen (200 mg/day) and Paracetamol (750 mg, three times per day) were administered for pain relief for three days after the surgeries. All implants were positioned 2 mm intra-bone (subcrestal) with a healing abutment, which in all cases was 1 mm outside the mucosa. After 30 days, the healing abutment was removed, and an impression was performed to install a provisional crown within 45 days after the surgery.

After the implant insertion, the ISQ value was measured using the Ostell Mentor device (Integration Diagnostics AB, Göteborg, Sweden) with a magnetic sensor type 49 (Smartpeg, Integration Diagnostics AB, Göteborg, Sweden), which was screwed into each implant and tightened to approximately 5 Ncm. The ISQ values were measured immediately after the implant insertion into the bone (T1—baseline) at 30 days (T2) and 45 days (T3) after surgeries. The measurements were obtained in two directions: buccolingual (B-L) and mesiodistal (M-D). Additionally, for the presentation of ISQ values evaluating the distribution of these data, an average was made between the measurements obtained in both directions (B-L and M-D). The scale provided by the device manufacturer (Osstell) was used to present the distribution and evaluation of the number of implants between each range of the ISQ values [26].

### 2.5. Statistical Analysis

Descriptive statistics (Mean, Median, SD, 95% CI, box plots) were generated for the insertion torque (ITV) and ISQ values for the DC and MAE groups. Furthermore, normality and equal variance steps were applied to determine the homogeneity of data distribution and to select the most appropriate parametric or non-parametric tests to investigate possible differences between DC and MAE for ITV and ISQ over time. The influence of implant macrogeometry (DC and MAE groups), location (maxilla and mandible), diameter (3.5 and 4.0 mm), length (9, 11, and 13 mm), and period (baseline, 30 days, and 45 days) to predict ITV and ISQ buccolingual and mesiodistal values were screened using multiple linear regression analysis. All the statistics were performed at the 5% significance level using dedicated software (SigmaStat for Windows version 4.0; Systat Software, Inc.). Graphs were generated using another program (MedCalc Statistical Software version 20.113 (MedCalc Software Ltd., Ostend, Belgium; https://www.medcalc.org; accessed on 1 December 2022).

Considering the two factors (implant design, arch type) as independent variables and the ISQ as the dependent variable, four groups are possible. The ANOVA sample size tool of SigmaStat provided the following: a minimum of 23 samples per group in the case of 8 ISQ points of difference while still considering a standard deviation value of 8 points. In the case SD values are 6 and the ISQ difference is 8, the minimum number per group would be 14 dental implants.

## 3. Results

**Figure 2:**
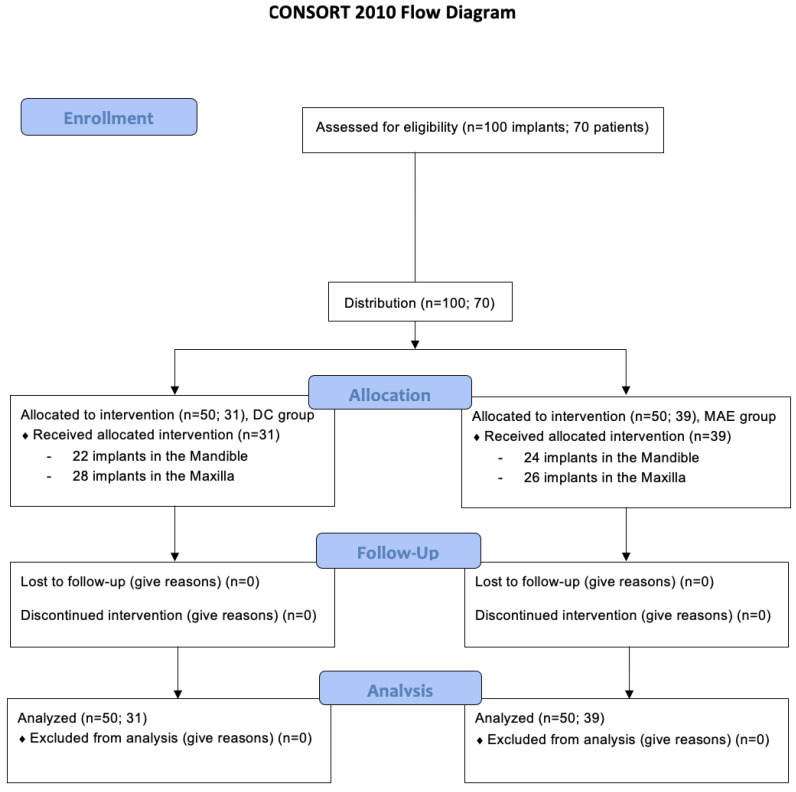
The CONSORT 2010 flow diagram.

All seventy patients who received 100 implants (*n* = 50 per group) had a typical postoperative course, free of complications and with a low rate of inflammation, as expected for this type of procedure. None of the implants showed signs of instability at the pre-determined times for evaluation. Detailed distributions for the groups regarding implant diameter, length, and the overall mean of ISQ values over investigated periods by arch type are depicted in Table 1. None of the patients in this study dropped out of treatment or missed pre-determined evaluation periods. Figure 2 shows the CONSORT 2010 flow diagram.

### 3.1. Insertion Torque Values

**Figure 3:**
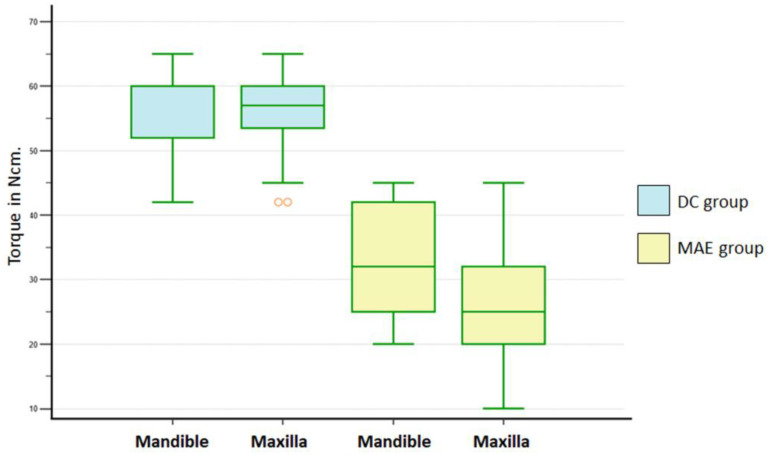
Box-plots graph (1st, 2nd, Median, 3rd, 4th quartiles) of ITV for DC and MAE groups within each arch. Outlier values are indicated in orange.

The 2-way ANOVA test (implant, arch) revealed statistically significant differences for implant macrogeometry (*p* < 0.001) and arch (*p* = 0.005). Furthermore, a significant interaction between implant macrogeometry and arch was detected (*p* = 0.013). The student-Newman-Kellus test was applied for multiple comparison procedures and revealed significant differences (Table 2). Figure 3 shows a graph of the data distribution.

### 3.2. ISQ Values

**Figure 4:**
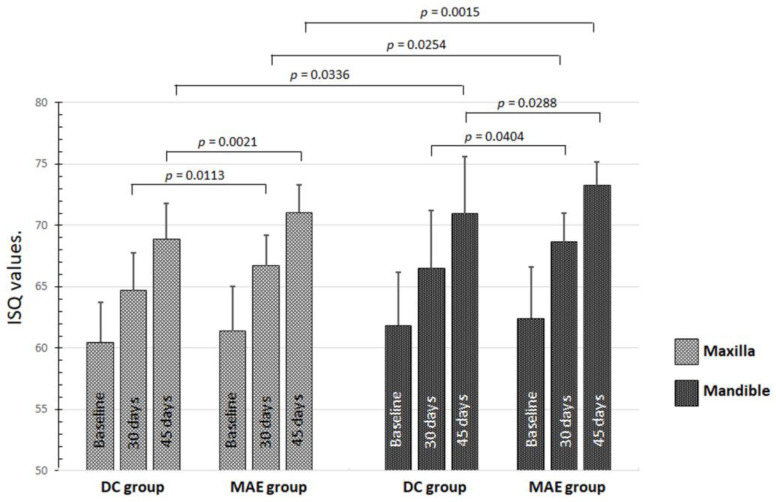
Graph of the data distribution and statistical differences of ISQ values inside and between both groups in maxilla and mandible on the 3 times.

The evaluation of the average ISQ between the values measured in both directions (B-L and M-D) for both groups (Table 1) showed a significant difference between the groups after 30 days (*p* = 0.0004) and 45 days (*p* = 0.0001). The MAE group showed higher values than the DC group. In the intragroup and intergroup comparisons between implants placed in the maxilla and the mandible, there were no statistical differences (*p* > 0.05) for ISQ values at T1 (baseline). However, at other periods, statistical differences were detected, as shown in the graph in Figure 4.

**Figure 5:**
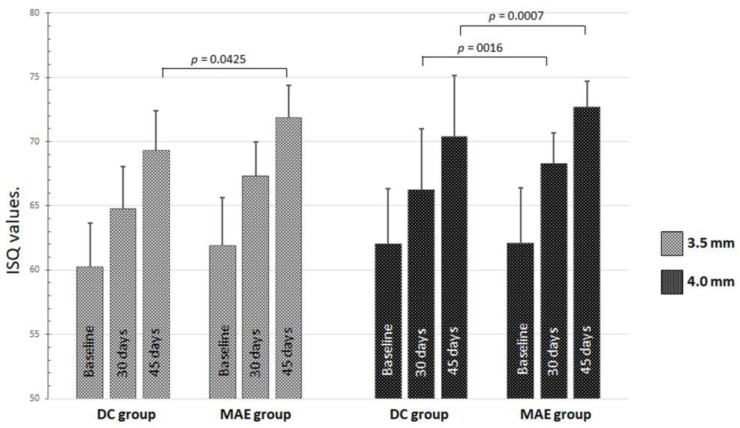
Graph of the data distribution and statistical differences of ISQ values inside and between both groups of implant diameters on the 3 times.

Regarding the comparisons of the diameter of the implants installed, regardless of the arch, few statistical differences were detected intragroup and intergroup in the 3 periods analyzed (Figure 5).

### 3.3. B-L ISQ Values of Mandible and Maxilla

**Figure 6:**
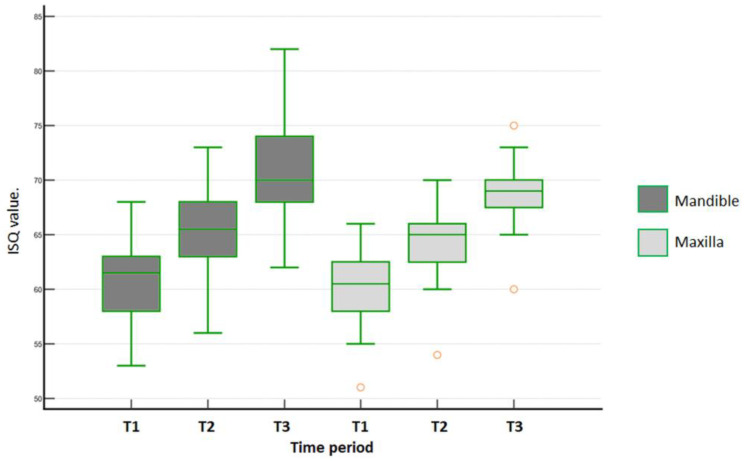
Data distribution of B-L ISQ values measured for DC group within each arch in each time period (T1 = baseline; T2 = 30 days; and T3 = 45 days). Outlier values are indicated in orange.

In the mandible for the DC group, the B-L ISQ values over time were statistically different (one-way ANOVA repeated measures, *p* < 0.001). Afterwards, Tukey’s test identified significant differences among all periods (*p* < 0.001). In the maxilla, B-L ISQ values for the DC group over time were statistically different (Friedman´s repeated measures on ranks, *p* < 0.001). Furthermore, all multiple pairwise comparisons (Tukey´s test) indicated differences across all tested periods (*p* < 0.001). Figure 6 shows the data distribution of the DC group for the mandible and the maxilla in the 3 periods of evaluation.

**Figure 7:**
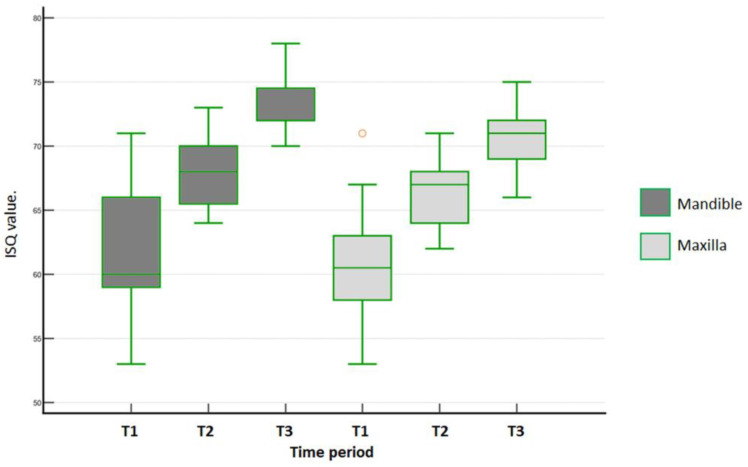
Data distribution of B-L ISQ values measured for MAE group within each arch in each time period (T1 = baseline; T2 = 30 days; and T3 = 45 days). Outlier values are indicated in orange.

In the mandible for the MAE group, the B-L ISQ values over time were statistically different (Friedman´s repeated measures on ranks, *p* < 0.001). Tukey´s test then identified the following significant differences: baseline × 30 days (*p* = 0.002), baseline × 45 days (*p* < 0.001). and 30 × 45 days (*p* = 0.002). In the maxilla, B-L ISQ values for the MAE group over time were statistically different (Friedman´s repeated measures on ranks, *p* < 0.001). Furthermore, all multiple pairwise comparisons (Tukey´s test) indicated the following differences: baseline × 30 days (*p* = 0.01), baseline × 45 days (*p* < 0.001), and 30 × 45 days (*p* < 0.001). Figure 7 shows the data distribution of the MAE group for the mandible and the maxilla in the 3 time periods of evaluation.

### 3.4. M-D ISQ Values of Mandible and Maxilla

**Figure 8:**
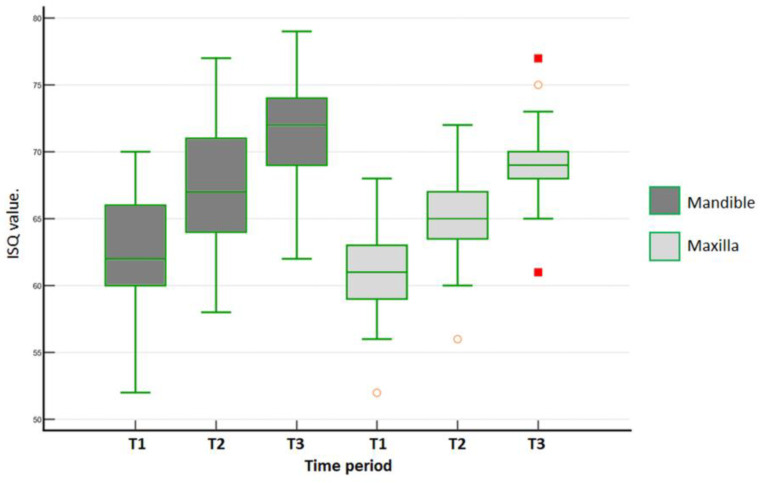
Data distribution of M-D ISQ values measured for DC group within each arch in each time period (T1 = baseline; T2 = 30 days; and T3 = 45 days). Outlier values are indicated in orange and red.

In the mandible for the DC group, the M-D ISQ values over time were statistically different (one-way ANOVA repeated measures, *p* < 0.001). Afterwards, the Tukey test identified significant differences among all periods (*p* < 0.001). In the maxilla for the DC group, the M-D ISQ values over time were statistically different (Friedman´s repeated measures on ranks, *p* < 0.001). The Tukey test identified differences among all periods (*p* < 0.001). Figure 8 shows the data distribution of the DC group for the mandible and the maxilla in the 3 time periods of evaluation.

**Figure 9:**
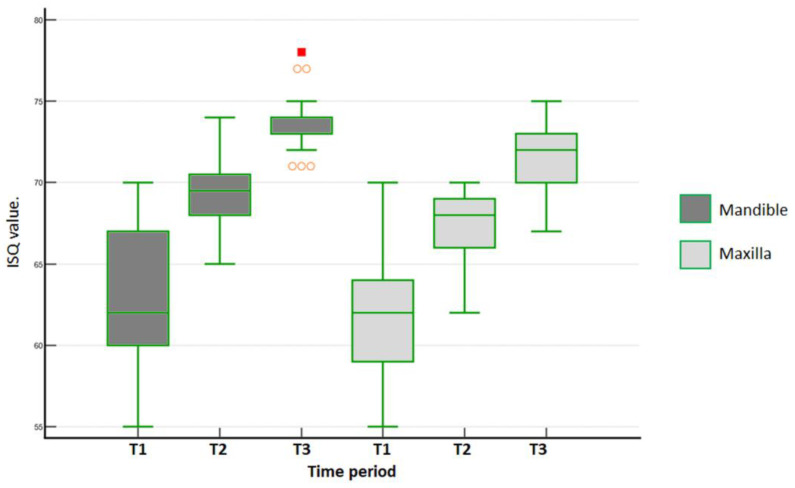
Data distribution of M-D ISQ values measured for MAE group within each arch in each time period (T1 = baseline; T2 = 30 days; and T3 = 45 days). Outlier values are indicated in orange and red.

Over time, the M-D ISQ values in the mandible for the MAE group were statistically different (one-way ANOVA repeated measures, *p* < 0.001). Afterwards, the Tukey test identified significant differences among all the periods (*p* < 0.001). In the maxilla, the M-D ISQ values for the MAE group over time were statistically different (Friedman´s repeated measures on ranks, *p* < 0.001). Furthermore, the Tukey test identified the following differences among periods: baseline × 30 days (*p* = 0.006), baseline × 45 days (*p* < 0.001), and 30 × 45 days (*p* < 0.001). Figure 9 shows the data distribution of the MAE group for the mandible and the maxilla in the three time periods of evaluation.

### 3.5. Multiple Linear Regression for Dependent Variable

The implant macrogeometry (DC and MAE groups), implant position (maxilla and mandible), diameter (3.5 and 4.0 mm), length (9, 11, and 13 mm), and periods (0, 30, and 45 days) were considered as independent variables. The *R-value* for IT was *R* = 0.871, and the one-way ANOVA test result was *p* < 0.001. The dependent variable insertion torque could be predicted from a linear combination of the independent variables: implant macrogeometry (*p* < 0.001) and implant position (*p* = 0.005). For B-L ISQ values, the *R-value* was *R* = 0.76, and the one-way ANOVA test result was *p* < 0.001. The dependent variable ISQ buccolingual could be predicted from a linear combination of the independent variables: implant macrogeometry (*p* < 0.001) and time (*p* < 0.001). Finally, for M-D ISQ values, the *R-value* was *R* = 0.76, and the one-way ANOVA test result was *p* < 0.001. The dependent variable ISQ mesiodistal could be predicted from a linear combination of the independent variables: implant macrogeometry (*p* < 0.001), implant position (*p* = 0.007), and time (*p* < 0.001).

### 3.6. Correlations between ITV and ISQ over Time

The correlation coefficients for each implant macrogeometry/arch according to the period are shown in Table 3.

## 4. Discussion

Adequate primary stability in the bone bed is essential for achieving osseointegration [2,5,6], which is directly dependent on bone quality, the surgical technique used, and the characteristics of the implant design [20]. Thus, the present clinical study compares the insertion torque and resonance frequency analysis of two implants with different macrogeometries placed into healed sites and evaluated at three different periods (0, 30, and 45 days). The clinical results obtained in this study showed that changes in the macrogeometry of implants could change their biomechanical behavior (IT and RFA values). Furthermore, the results obtained agree with other studies carried out in vitro by our research group comparing the same implant models [16], in which significant differences were observed in the insertion torque values but not in the initial stability (RFA). However, clinical studies in patients are essential to prove the effectiveness of any material. Furthermore, in order to keep the peri-implant health condition, mechanical debridement therapy was periodically recommended to the patients, avoiding peri-implant diseases, keeping the stabilization of the surrounding hard and soft tissues [27], which can also have influence by the type of implant [28]. In addition, if necessary, the adjunctive use of ozone, glycine/erythritol, probiotics, and chlorhexidine treatment may be considered [29].

Previous studies showed that conical implants have higher primary stability values than cylindrical implants [30,31,32]. Two implants with the same conical design were then compared in our research. Furthermore, the type of osteotomy protocol used in our clinical trial was the same for the two implant models, strictly following the protocol recommended by the manufacturer. As reported by other authors [30], conical implants with conventional designs, similar to those used in the DC group due to their tapered-screw shape, could provide higher insertion torque because they exert pressure on the bone at the time of installation, a fact corroborated by our study. However, the implants used in the MAE group, with healing chambers in their bodies and despite having conical shapes, had significantly lower insertion torque values than the implants in the DC group: in the maxilla, the MAE group presented an average value that 122% lower than that of the CD group, and in the mandible, the difference was 68.7% (DC > MAE). Other authors have reported that implants inserted with less torque may cause less bone trauma and facilitate the osseointegration process [5,6,33]. In addition, recent preclinical studies have shown that implants with a macrogeometry that decreases insertion torque without affecting their primary stability can produce less marginal bone loss [34].

In the multiple linear regression considering the insertion torque as a dependent variable, statistical differences were detected for the implant macrogeometry (DC and MAE groups) and implant position (maxilla and mandible), corroborating results obtained in other clinical studies [35,36,37]. Clinically, the assessment of stability and osseointegration of implants is achieved through radiographs, probing of the implant to assess possible mobility or sensitivity and percussion tests. However, these assessments have limitations regarding standardization and differences between the professionals who perform them [38]. Currently, a non-invasive method has been developed to facilitate and standardize the measurements and analysis of the stability of implants in their different phases, such as resonance frequency analysis (RFA), which has been widely used in different in vitro and in vivo studies [2,3,5,16]. The RFA technique has been increasingly incorporated into the clinical routine because it is easy to perform and does not present any risk or discomfort to the patient. In addition, studies have shown that the measurement of implant stability by RFA showed a strong correlation with histomorphometric measures, showing the evolution of bone healing around the implant can be analyzed by this method [39,40].

Implant dimensions (diameter and length) can significantly influence initial stability values, especially in low-density bone [41]. Some authors have suggested that longer and wider implants can increase primary stability due to the increased bone-implant contact surface area and achieve significantly higher ISQ values [42,43]. In the present study, implants with different lengths (9, 11, and 13 mm) and two diameters (3.5 and 4.0 mm), with two different macrogeometries, revealed no significant differences in ISQ values between the implant dimensions tested. This result corroborates the results found in other studies, which reported no statistically significant differences in ISQ due to length or diameter [20,44,45].

During the early period, predating the evaluations through RFA, the ISQ value varied in both groups. However, at baseline (immediately after the insertion of the implant), the overall mean of the ISQ was 61.1 ± 3.78 for the DC group and 61.9 ± 3.92 for the MAE group, without a statistical difference (*p* = 0.4986). These values indicate good primary stability, with values within the range indicative of adequate for obtaining osseointegration [45,46,47]. Clinical studies have shown that most implants installed in areas of healed bone, both in the maxilla and mandible, have mean ISQ values above 60 in most cases, with higher values for the mandible [25,45,48,49]. Our results showed the ISQ average values for maxilla of 60.5 ± 3.25 for the DC group and 61.4 ± 3.67 for the MAE group (*p* = 0.2246) and the mandible of 61.9 ± 4.33 for the DC group and 62.4 ± 4.19 for the MAE group (*p* = 0.8862). In all situations highlighted, the ISQ values obtained in this study using two different macrogeometries were within the range of reported values in the previously mentioned studies. Furthermore, we observed that the mean values increased significantly in the evaluations performed after 30 and 45 days.

Regarding the direction of measurements by RFA (buccolingual and mesiodistal), other studies that evaluated this variable as a possible important clinical parameter reported that no significant differences were found between ISQ values [25,45,48], corroborating the results obtained in our study. Although the statistical analyzes carried out with the data obtained in both directions (B-L and M-D) showed statistically non-significant values, these were presented to reinforce the scientific basis for this parameter. However, in cases where the amount of bone around the implant is not uniform, for example, where there is a lack of any bone wall or implants inserted into fresh sockets (after extraction), measurements in different directions and the results should be considered [50]. In the multiple linear regression, considering the buccolingual ISQ as a dependent variable, statistical differences were detected for the implant macrogeometry (DC and MAE groups) and periods (0, 30, and 45 days), while for the mesiodistal ISQ, the implant macrogeometry, implant position (mandible and maxilla), and time showed statistically significant differences. These results were similar to those obtained in other studies [30,44,48].

Regarding the macrogeometry of the implants studied, the results showed that this is an important factor to be considered when accelerating the healing process, corroborating the pre-clinical studies recently published by our research group [2,34]. Finally, the correlation analysis between the insertion torque and the measured stability values (ISQ) showed no correlation between these two parameters, corroborating studies by other authors [16,44,51,52].

Among the limitations of this study, we can mention that the results obtained are not automatically applied to implants with other macrogeometries since each implant design or dimension (length and diameter), as well as the osteotomy protocol used, must be evaluated separately. On the other hand, implants were placed in different patients and areas of the maxilla and mandible without following a single standard of quality and quantity for the residual bone tissue.

## 5. Conclusions

Modifications of the implant’s macrogeometry, by adding a healing chamber to its design (MAE group), produced a significant reduction in the insertion torque of the implants compared with the DC group, which presented a conventional implant design (without healing chambers). However, this reduction in ITV did not decrease the initial stability of these implants as measured by RFA. In addition, the stability measurement 30 and 45 days after implant placement showed higher values for the MAE group than those obtained for the CD group. Finally, both implant models tested showed good evolution of the ISQ values (stability) in the early evaluation period for osseointegration proposed in this study.

## Figures and Tables

**Table 1:** Demographic data.

	Surgical Site Condition					
	DuoCone (DC Group)			Maestro (MAE Group)		
Number of patients (total)	31			39		
Number of implants (total)	50			50		
Maxilla (per implant)						
diameter						
3.5 mm	7			5		
4.0 mm	21			21		
Length						
9 mm	2			10		
11 mm	17			14		
13 mm	9			2		
Mandible (per implant)						
diameter						
3.5 mm	15			16		
4.0 mm	7			8		
Length						
9 mm	20			21		
11 mm	2			3		
13 mm	0			0		
Maxilla (ISQ values)	T1	T2	T3	T1	T2	T3
Mean	60.5	64.7	68.9	61.4	66.7	71.1
SD	3.25	3.09	2.88	3.67	2.51	2.27
no. of implants ISQ ≤ 60	13	2	0	10	0	0
no. of implants ISQ 60–65	14	15	2	12	7	0
no. of implants ISQ 65–70	1	10	20	3	19	8
no. of implants ISQ > 70	0	1	6	1	0	18
Mandible (ISQ values)	T1	T2	T3	T1	T2	T3
Mean	61.9	66.5	71.0	62.4	68.7	73.3
SD	4.33	4.71	4.65	4.19	2.33	2.94
no. of implants ISQ < 60	6	2	0	8	0	0
no. of implants ISQ 60–65	11	6	3	7	2	0
no. of implants ISQ 65–70	5	9	5	8	18	0
no. of implants ISQ > 70	0	5	14	1	4	24

ISQ: Implant stability quotient; T1: baseline; T2: at 30 days; T3: at 45 days.

**Table 2:** Insertion torque values by groups.

	DuoCone		Maestro	
	Maxilla	Mandible	Maxilla	Mandible
Mean	56.21 ^a^	56.72 ^c^	25.30 ^a,b^	33.62 ^b,c^
SD	6.27	6.71	8.58	8.97
95% CI	53.78–58.64	53.75–59.60	21.83–28.77	29.83–37.41

Same uppercase letters indicate statistically significant differences intergroup and/or intragroup: a, b, c (*p* < 0.001).

**Table 3:** Pearson correlation of ITV and ISQ over time.

Insertion Torque	ISQ Bucco-Lingual			ISQ Mesio-Distal		
	Baseline	30 Days	45 Days	Baseline	30 Days	45 Days
ITV DC maxilla	0.32	0.34	0.34	0.35	0.35	0.36
ITV DC mandible	−0.07	0.008	−0.14	−0.03	−0.07	−0.1
ITV MAE maxilla	0.24	0.16	−0.01	0.27	0.01	−0.04
ITV MAE mandible	−0.12	−0.06	−0.22	−0.10	−0.22	−0.08

## Data Availability

All data generated or analyzed during this study are included in this published article.
